# Visualizing diastolic failure: Non-invasive imaging-biomarkers in patients with heart failure with preserved ejection fraction

**DOI:** 10.1016/j.ebiom.2022.104369

**Published:** 2022-11-21

**Authors:** Alexander Schulz, Andreas Schuster

**Affiliations:** Department of Cardiology and Pneumology, Georg-August University, University Medical Center Göttingen, Göttingen, Germany

**Keywords:** HFpEF, Diastolic dysfunction, Non-invasive imaging, Imaging markers

## Abstract

Heart failure with preserved ejection fraction is an increasing challenge for modern day medicine and has been drawing more attention recently. Invasive right heart catheterization represents the mainstay for the diagnosis of diastolic dysfunction, however due to its attributable risk of an invasive procedure, other non-invasive clinical pathways are trying to approach this pathology in clinical practice. Diastolic failure is complex, and imaging is based on various parameters. In addition to transthoracic echocardiography, numerous novel imaging approaches, such as cardiac magnetic resonance imaging, computed tomography, positron emission (computed) tomography or single photon emission tomography techniques are being used to supplement deeper insights into causal pathology and might open targets for dedicated therapy options. This article provides insights into these sophisticated imaging techniques, their incremental value for the diagnosis of this poorly understood disease and recent promising results for an enhanced prognostication of outcome and therapy monitoring.

## Background

Heart failure is a significant and well-known challenge in modern day medicine. Particularly the subgroup with preserved ejection fraction is underrecognized and treatment of underlying etiologies often delayed. It accounts for about 50% of heart failure^1^ while being responsible for even more than half of the admissions to hospital due to heart failure.^2^ The associated mortality is 8-times higher compared to normal cardiac function and after the development of symptomatic heart failure for the first time, the 6-month mortality is similar to heart failure with reduced ejection fraction (HFrEF).^3^ Furthermore, the 5-year mortality is higher than the one associated with many forms of cancer^1^ and the affiliated socio-economic costs reflect one of the largest societal problems in modern healthcare.^3^

While resuming the indisputable clinical impact of heart failure with preserved ejection fraction (HFpEF) the important question arises: why was it such an underrecognized disease with lacking attention until lately?

Going back to the “older brother” HFrEF, the quantification of the ejection fraction (EF) with transthoracic echocardiography (TTE) is a straightforward method to make evidence-based assumptions on the stage and outcome of the disease.^4^ Moreover, it leads to further potentially necessary, and guideline-recommended diagnostic and therapeutic consequences.^4^

On the other hand, the diagnosis of HFpEF requires multiple steps including pretest assessments and verifying diagnostic tests as soon as it is suspected. Therapeutic consequences are rare^5^, ^6^, ^7^ which partly contributed to limited efforts in this research field.

In practice, two scoring systems are suggested as the most practical approaches for diagnosing HFpEF: The H2FPEFF score (heavy, 2 or more hypertensive drugs, atrial fibrillation, pulmonary hypertension, elder age >60, elevated filling pressures^8^) and the HFA-PEFF score (Heart Failure Association pretest assessment, echocardiography and natriuretic peptide, functional testing, final aetiology^9^) as American and European equivalent of each other. Both include multiple echocardiographic measures, laboratory markers as well as patient characteristics and comorbidities to finally strengthen the suspicion. While the comparison of both scores showed conflicting evidence on superiority^10^^,^^11^ still, both do not compete with right heart catheterization (RHC) as the invasive reference standard for the final diagnosis.^9^^,^^12^

The complex nature of current diagnostic procedures might arise from the lack of the pathophysiological understanding of the disease. However, as HFpEF is drawing an increasing attention in research work, more innovative and non-invasive approaches arise to aid its diagnosis and monitoring.

## Pathophysiology

This review focusses on non-invasive imaging biomarkers in HFpEF and their application in clinical routine and research. However, it is important to introduce pathophysiological basics of HFpEF for a better understanding of the imaging markers.

### HFpEF—A distinct type of heart failure?

Literature discusses controversially if HFrEF and HFpEF are distinct or overlapping phenotypes of heart failure as both share common signs and symptoms. Considering just the EF, even for patients diagnosed with HFpEF, specific sub-phenotypes with an EF between 50–60% and >60% were found recently.^13^

From a pathophysiological point of view, HFrEF is mostly caused by myocardial damage e.g., infarction, inflammation, toxins, or genetic disposition with a subsequent loss of cardiac output. HFpEF on the other hand is mostly resulting from systemic dysregulation which later leads to loss of function.^14^^,^^15^ In fact, just a minority of patients with HFpEF can be associated with a specific cardiac diagnosis like hypertrophic, infiltrative cardiomyopathy or constrictive pericarditis.^16^ Most commonly patients will have a history of common cardiac risk factors causing a pleiotropic remodeling or myocardial hypertrophy.^16^

### Substrates and risk factors of HFpEF

Diastolic dysfunction was already observed to be strongly associated with aging in previous studies, as ventricular filling decreases with age in normal subjects. The aging process mainly contributed to regional diastolic asynchrony with an impairment of rapid filling phases.^17^ However, besides advanced age additional common cardiac risk factors like components of the metabolic syndrome, such as diabetes mellitus and obesity, were associated with important remodeling processes in HFpEF.^16^ In particular, arterial hypertension (aHT) is referred as a major substrate to HFpEF.^1^^,^^18^ While aHT is known to reduce vessel compliance, it also has been shown to be associated with concentric left ventricular (LV) remodeling and a consecutive loss of ventricular compliance.^19^^,^^20^ All risk factors outlined tend to produce oxidative stress inducing a proinflammatory/profibrotic state. A subsequent remodeling of the myocardium and a simultaneous stimulation of myocardial hypertrophy can be observed.^21^, ^22^, ^23^ The evidence of the shared inflammatory genesis of the mentioned determinants is supported by the observation of HFpEF in patients with COVID-19 with similar levels of inflammatory transmitters both in COVID-19 and non-COVID-19 HFpEF groups.^24^

An important interdependence can be observed between HFpEF and atrial fibrillation (AF).^25^ The co-existence of both pathologies, AF and HFpEF, often results in lower exercise tolerance and an earlier clinical appearance.^26^ AF is not only one of the primary predictors of HFpEF, they both share left atrial (LA) remodeling as a major pathogenetic mechanism.^27^ Since this remodeling is mainly triggered by atrial fibrosis, the increased atrial stiffness explains why, even with lower LA volumes, the LA peak pressure is significantly higher compared to patients with HFrEF.^25^

While AF is most likely a consequence to the increased LV filling pressures with subsequently increased pressures and dilatation of the atrium,^25^ other theories hypothesize, that increased heart rates might as well trigger LV fibrosis which in turn results in HFpEF.^26^^,^^28^ Even if the latter theory only has little evidence, the temporal sequence of the occurrence of AF and HFpEF has yet to be discovered and might lead to further pathophysiological insights.^27^

## The diastolic (dys)function of the heart

Imaging in HFpEF is mainly based on visualizing the decompensation of the diastolic cardiac function and the subsequent consequences.

During a regular cardiac cycle, the diastole is subdivided into major phases including: 1. relaxation of the LV and a base-ward movement of the mitral annulus to fill the LV with blood; and 2. the atrial contraction to guarantee augmentation of ventricular filling in late ventricular diastole. While the first phase during the early diastole is an active mechanism of LV myocytes and biochemical events to initiate ventricular relaxation, the second phase during late diastole is a passive property of the LV, actively contributed by the LA.^29^

Finally, during the ventricular systole, the atrial relaxation initiates a passive collection of the pulmonary venous return into the atrium to initiate another diastolic cycle.^30^

**Fig. 1:**
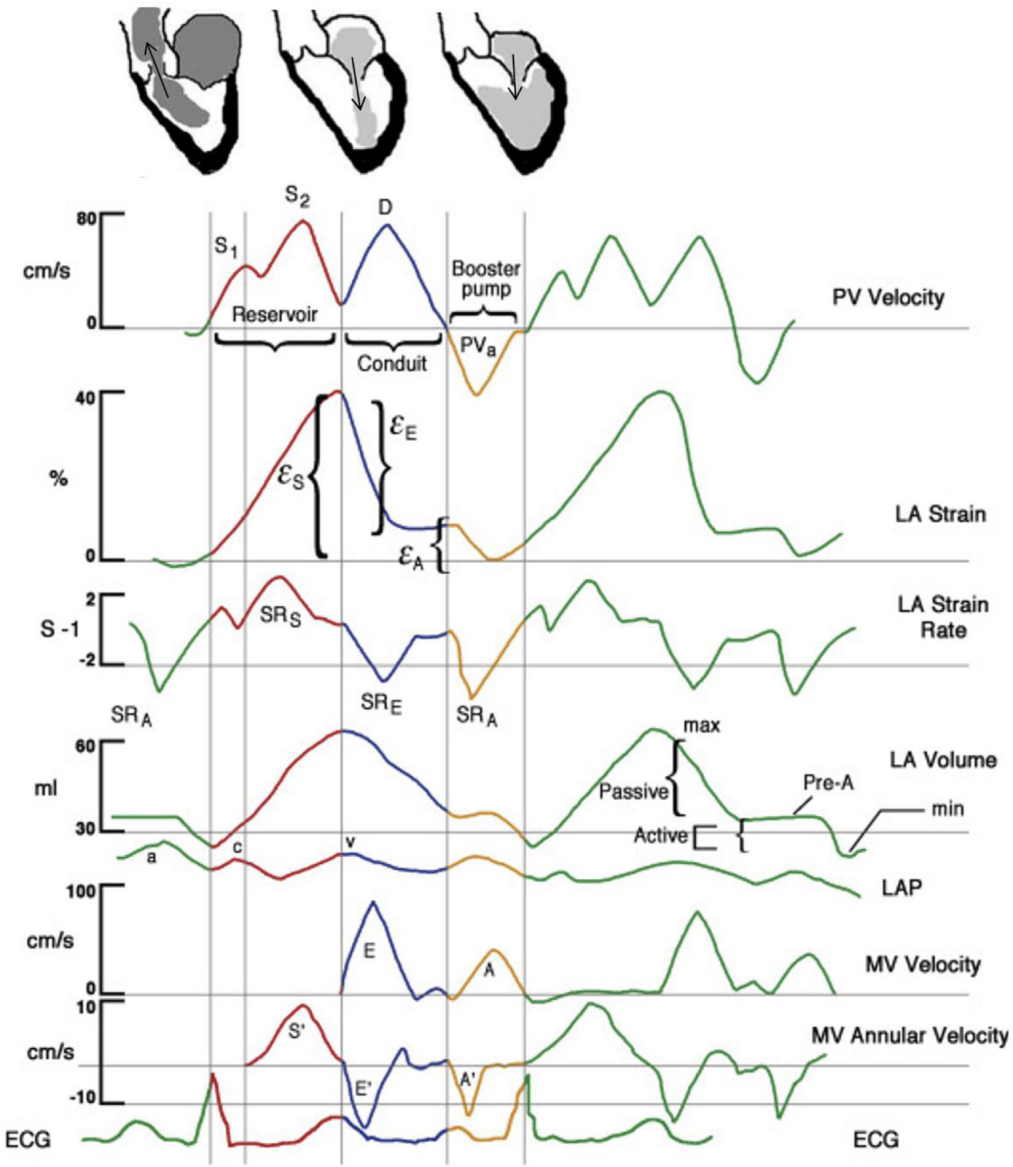
**Functions of the Left Atrium within the cardiac cycle**. Pulmonary venous (PV) velocity, left atrial (LA) strain (ε), LA strain rate (SR), LA volume, left atrial pressure (LAP), and mitral spectral and tissue Doppler. ECG = electrocardiogram; MV = mitral valve. Reservoir (red), conduit (blue), and booster pump (yellow) functions are additionally illustrated by schematic drawings at the top (Modified after Mehrzad et al. 2014,^31^ © CC-BY 3.0 http://creativecommons.org/licenses/by/). The rest of the figure was illustrated by Craig Skaggs, Hoit 2014^30^ ©2022 with permission from Elsevier.

Those three phases are markers of a specific cardiac function as measures of compliance and contractility^30^^,^^32^: Atrial reservoir function (gathering of venous return during ventricular systole) is a marker of atrial compliance, atrial conduit function (traverse of blood to the ventricles during early ventricular diastole) a surrogate marker for LV compliance and atrial booster pump function (increment of ventricular filling in late ventricular diastole following the active atrial contraction) a marker of atrial contractile reserve (see Fig. 1).^32^^,^^33^

In diastolic dysfunction, impaired LV relaxation and stiffness determine impaired atrial conduit function, which induces an increase of the atrial booster pump function to compensate for the decreased LV filling. This is paralleled by a mismatch of both diastolic phases i.e., the ventricular influx by the atrial contraction catches up with the influx by the atrial conduit function. The incremental atrial workload secondary to the ventricular diastolic disfunction can result in atrial decompensation. Hand in hand with the failure goes atrial dilatation and an absolute reduction of the atrial stroke volume, leaving by implication, a reduced ventricular influx during diastole.^34^^,^^35^ As this compromises blood flow through the heart, the ventricular outflow is consequently reduced. With consistent inflow the increasing intraventricular pressure leads to secondary backward congestion to the lungs, and often to the first symptoms of the disease to the patient as dyspnea.^12^^,^^36^

## Clinical implications and definition of HFpEF

Patients suffering from HFpEF are characterized by their inability to adequately react to physical exercise due to an impaired atrial contractile reserve.^37^ During RHC as the diagnostic reference standard, this is paralleled by the increase of the pulmonary capillary wedge pressure (PCWP) as a marker of acute left heart failure during physiological exercise.^12^ The PCWP is used as an indirect parameter of LA pressure and is measured following the inflation of a balloon attached to the pulmonary artery catheter. This allows to estimate LA pressure through the conduit of the pulmonary capillary system but requires accurate complete wedging, the absence of abnormalities of the pulmonary microvascular system and confounders between the pulmonary venules and the LA.^38^

HFpEF is defined as a PCWP ≥25 mmHg during exercise,^12^ whereas normal values for PCWP during supine exercise were described <25 mmHg in earlier studies.^39^, ^40^, ^41^ Interestingly, patients with pathologic PCWP under exercise stress had normal filling pressures at rest, were euvolemic and had normal (nt-)proBNP values.^12^

The observation of increased PCWP during exercise stress was a milestone in the diagnosis of HFpEF and allows the identification of an otherwise clinically inapparent disease for the first time.

As RHC is an invasive procedure, other diagnostic pathways have been explored for an early diagnosis in clinical routine.

The most frequently used pathways are based on scoring systems consisting of clinical, laboratory and echocardiographic measurements to estimate the probability of HFpEF, the H2FPEF-Score^8^ and the HFA-PEFF Score.^9^

Both scores include parameters such as bodyweight, aHT, age and AF as well as distinctive echocardiographic parameters.

In case of uncertainty with e.g., intermediate scores functional testing with stress-echocardiography on the non-invasive side, or with RHC on the invasive side is recommended.

As soon as the diagnosis is established, an etiological work-up is necessary referring to a variety of diagnostic methods such as cardiac magnetic resonance imaging (CMR), biopsies, computed tomography (CT), positron emission computed tomography (PET-CT), single photon emission tomography (SPECT) or genetic testing. Only by the use of those advanced imaging- and diagnostic methods—HFpEF-like syndromes with advanced targeted therapy options can be identified and treated.^9^

## Guideline-recommended non-invasive imaging techniques

### Transthoracic echocardiography

Echocardiography must be highlighted first, as it is included in both above-mentioned scoring system and the primary approach to screen for a preserved EF. Starting with structural parameters there is the left ventricular mass index (LVMI) as a direct measure of myocardial hypertrophy with a consecutive thickening of the measured walls. The left atrial volume index (LAVI) is assessed in two orthogonal views and indicates atrial size.^42^ Additionally, the systolic pulmonary artery pressure (PAPsys) can be measured in echocardiography to indicate pulmonary hypertension.^43^^,^^44^ As it is measured by the velocity of the tricuspid regurgitation (TR) jet, this parameter is just a vague estimation and prone to error.

**Fig. 2:**
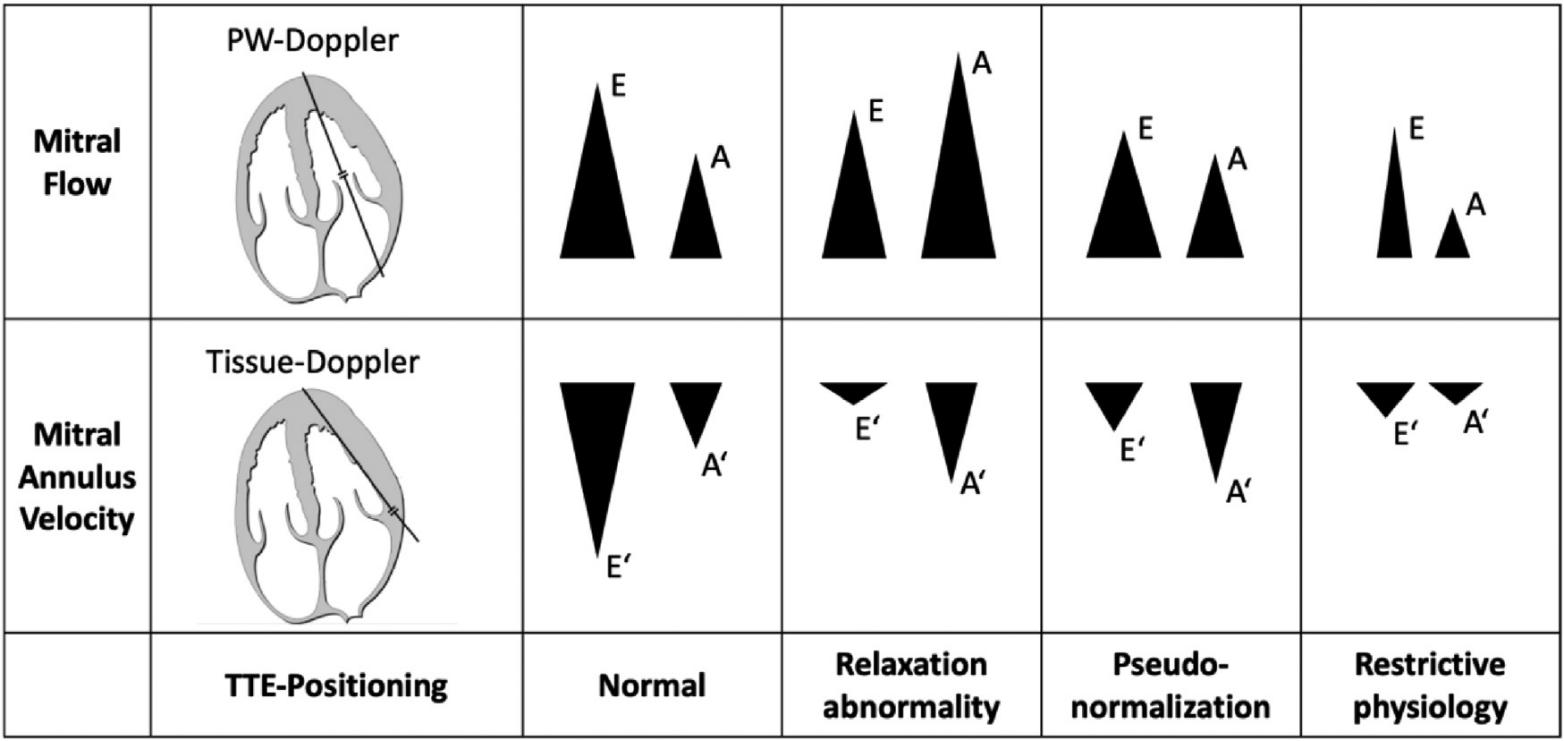
Patterns of the E, A, E′, and A′ Doppler waves during the development of left ventricular diastolic dysfunction as described by Sohn et al.^34^ Additionally, the positioning of the doppler probe within the apical 4-Chamber view during echocardiography is displayed. PW = Pulse-Wave, TTE = Transthoracic echocardiography. (Graphic of the 4-Chamber view modified after Patrick J. Lynch © CC-BY 2.5 http://creativecommons.org/licenses/by/).

An enhanced echocardiographic assessment of diastolic function should include the inflow through the mitral valve (E) and the inflow due to the contraction of the atrium (A) as indirect measures of the atrial conduit and booster pump function. Furthermore, the velocity of the mitral annulus (e′) should be measured in the septal and lateral region. In addition to the individual parameters, the derived ratios (E/e′, E/A) are important for the diagnosis and monitoring of HFpEF (see Fig. 2).

All illustrated functional parameters are measured by pulse-wave and tissue doppler signals over the mentioned structures. Those flow-derived echo parameters depend on a correct alignment of the ultrasound beam with the flow direction, which can be difficult, even for experienced echocardiographers.^45^ Apart from this, e′ as a measure of the lengthening of the LV is dependent on LV preload.^46^^,^^47^

**Fig. 3:**
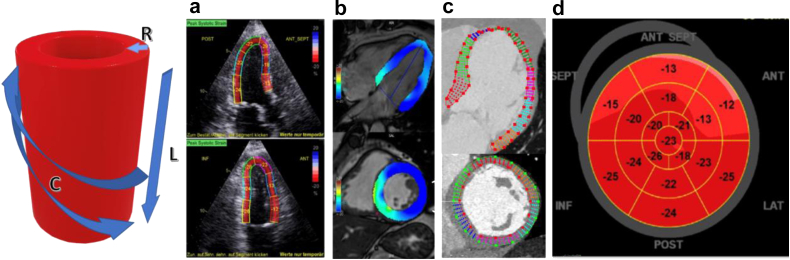
Cardiac deformation imaging with different modalities in an example of the left ventricle. The graphic on the left picturizes the directions of myocardial shortening within the different layers of the myocardium. The endocardial layer is formed by fibers with longitudinal (L) orientation. The mid-wall has a radial (R) orientation, while the epicardium is circumferentially (C) orientated. Using different imaging modalities, the shortening along the three directions can be visualized. The calculations are based on the tracking of individual pixels within a cine projection of the cardiac cycle. This method can be performed in a similar way for the other chambers of the heart. (a) Echo-strain imaging (Uhe et al. 2020,^53^ © CC-BY 4.0); (b) CMR-feature tracking (Scatteia et al. 2017,^54^ © CC-BY 4.0) and (c) CT-strain imaging (Vach et al. 2021,^55^ © CC-BY 4.0). (d) shows an example of a Bulls‘ eye trajectory of the myocardium with the relative shortening (%) for each region of the myocardium (Uhe et al. 2020,^53^ © CC-BY 4.0). https://creativecommons.org/licenses/by/4.0/.

A non-angle-dependent functional equivalent and innovative method to monitor LV shortening is multidimensional global longitudinal strain (GLS) using speckle tracking (compare Fig. 3).^48^ Reduced GLS is not only shown to predict cardiovascular outcome and prognosis^49^^,^^50^ but also correlates with invasively measured LV stiffness.^51^^,^^52^

As outlined before, exercise stress testing is an important element of the diagnosis of HFpEF and is mostly recommended in cases of uncertainty.^9^ In exercise stress-echocardiography the change of the TR velocity and E/e′ are measured. Both have been shown to indicate an increase of mean PCWP and pulmonary artery systolic pressure in patients with HFpEF.^42^^,^^56^ However, stress-echocardiography is still prone to errors under limited acoustic windows or suboptimal imaging quality due to breathing or physical motion during exercise.^56^

### Cardiac magnetic resonance, computed tomography, positron emission computed tomography and Scintigraphy

At the moment, CMR is being recommended for the detailed etiological work-up after establishing the diagnosis using the mentioned guidelines.^9^ This step should rule out for example storage diseases, acute and chronic inflammatory cardiomyopathies or genetic disorders associated with muscular dystrophy to guide them to specific diagnostic and therapeutic implementations.^9^

**Fig. 4:**
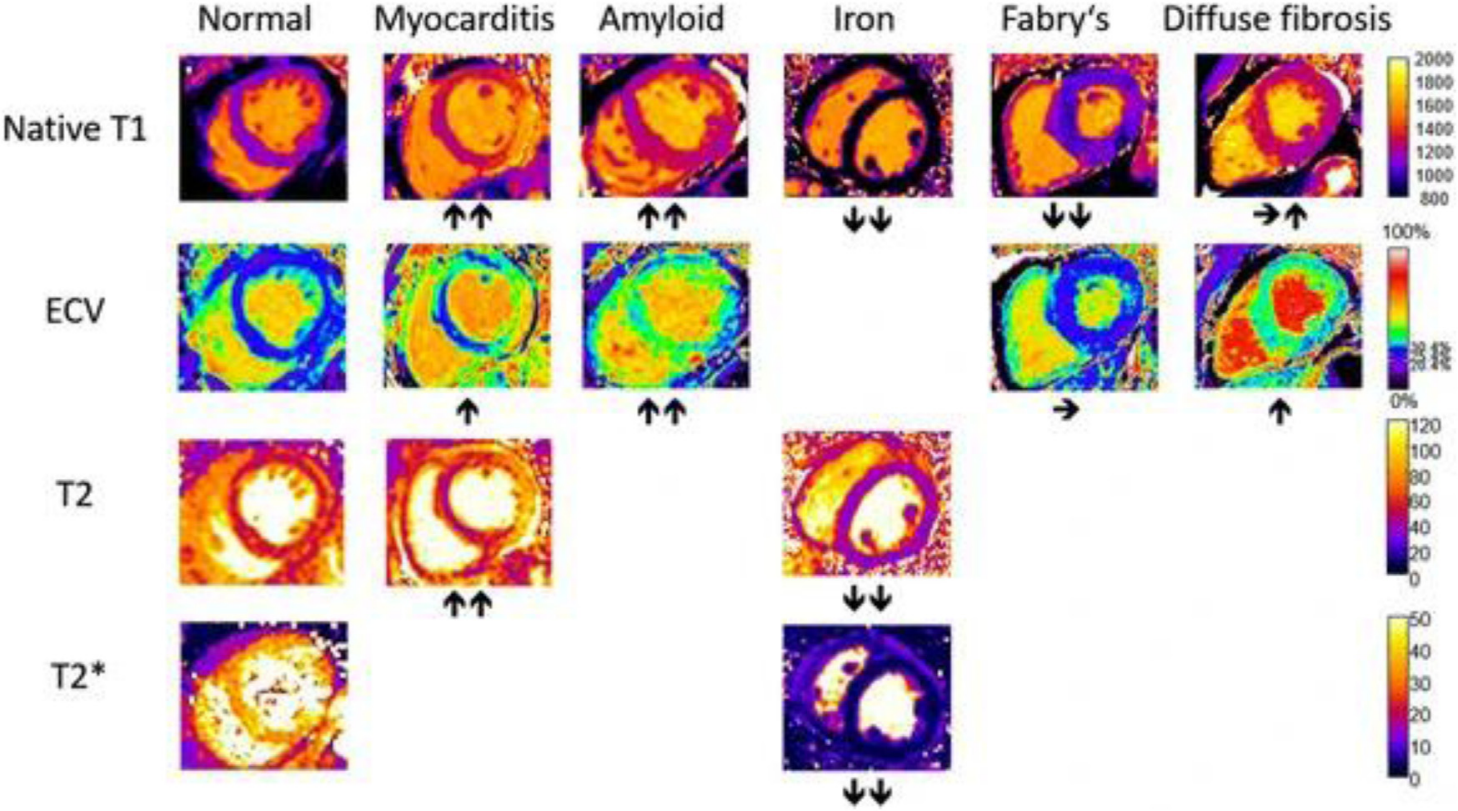
Typical patterns of CMR T1, T2, T2∗ and ECV Maps in different diseases. Those sequences and patterns can be used for detailed tissue differentiation and the diagnosis of distinct diseases. The arrows are indicating the relive change of the parametric maps compared to normal. By Messroghli et al., 2017^63^ © CC-BY 4.0 https://creativecommons.org/licenses/by/4.0/.

As used for various other diseases already, CMR-based tissue differentiation allows for the quantification of myocardial fibrosis by calculating the extracellular volume fraction (ECV) using native and contrast-enhanced quantitative T1 mapping sequences^57^ (see Fig. 4). This feature is of incremental value in HFpEF as the degree of LV stiffness and amount of myocardial fibrosis represents one of the key pathophysiological mechanisms. Furthermore it plays a major role in the diseases’ progression^58^^,^^59^ and is known to be associated with poor outcome.^60^

For the work-up of other cardiomyopathies, guidelines recommend further enhanced diagnostic imaging. This includes ^(99m)^Tc-DPD Scintigraphy for the identification of cardiac amyloidosis^9^, CT or PET-CT to rule-out ischemic heart disease and coronary artery stenosis using state of the art CT coronary angiography with calculation of the CT fractional flow reserve.^61^^,^^62^

Even though those imaging approaches are an indispensable part of the diagnostic work-up, their representation in current guidelines is rather small as some advances still need further validation while the increased costs compared to TTE cannot be disregarded. However, those innovative techniques using multimodal imaging show promising results for the initial diagnosis of HFpEF and are an important area of research to combine the ability of tissue characterization and diagnostic testing within one modality.

## Advances in imaging—How to detect and quantify

Multiple non-invasive imaging techniques improve the understanding of the underlying pathophysiology in HFpEF. This paragraph aims to give a brief overview on some of those methods, and their potential use in the clinical routine (compare Table 1)

**Table 1:** Non-invasive imaging markers in HFpEF.

Modality		Imaging marker	Displayed structure/function
**Non-invasive imaging tests for HFpEF as mentioned by current guidelines**^4^^,^^9^			
**TTE**		LV diastolic function	Mitral inflow, atrial outflow, myocardial shortening
		Myocardial morphology	LA/LV dimensions
		PAPsys	TI signal, pulmonary hypertension
**CMR**		Volumetry	Cardiac dimensions and function
		Tissue characterization and mapping	Tissue visualization (e.g. edema/fibrosis/remodeling)
**Specific tests**	**SPECT**	^(99m)^Tc-DPD uptake	ATTR-Amyloidosis
	**CT**	CT-angiography	Coronary artery disease
**Innovative non-invasive imaging markers**			
Imaging of cardiovascular flow, function, and kinetics			
**CMR/CT**		Strain/FT	Myocardial function/contractility
		Pulse wave velocity	Arterial stiffness
		4D flow	Kinetic energies
Cardiac perfusion, metabolism, and innervation			
**PET/CMR**		Coronary/Myocardial Perfusion	Microvascular disease
		Immunometabolism/Macrophages	Inflammation
**SPECT**		^123^I MIBG SPECT	Sympathetic innervation
**PET**		FDG-PET	Tissue viability, LA tissue function
Extracardiac structures			
**CMR/(CT)**		Epicardial adipose tissue	Epicardial fat
		Lung water density	Pulmonary congestion

TTE = transthoracic echocardiography; HFpEF = heart failure with preserved ejection fraction; PAPsys = pulmonary artery systolic pressure; LA = left atrium; TI = tricuspid insufficiency; FT = feature tracking; CMR = cardiac magnetic resonance imaging; LV = left ventricle; CT = computer tomography; PET = positron emission tomography; SPECT = single photon emission computed tomography.

### Imaging of cardiac function, vascular flow, and kinetics

#### Strain and feature tracking

At the present time CMR represents the reference standard for the assessment of myocardial systolic function due to its inherent superiority over TTE resulting from the better endocardial surface definition and its independency from acoustic windows.^64^ However, the interpretation of CMR-derived results is highly dependent on operator experience and can be maximized using quantitative deformation imaging such as CMR myocardial feature tracking (CMR-FT) as an equivalent to echocardiographic strain-imaging.^65^ CMR-FT based deformation imaging has been well validated^66^^,^^67^ whilst ventricular deformation imaging allows detailed assessments of systole (myocardial torsion, strain) and diastole (diastolic recoil, strain rate) and the assessment of atrial deformation offers precise quantification of the three phases of atrial physiology.^68^ Equivalent technical developments have been made in other imaging modalities like CT to assess both, volumetric and functional parameters of both ventricles^69^ and have been shown to be reproducible.^55^^,^^70^ CT-derived strain measurements provide a valuable alternative for patients with contraindications for CMR.^71^ Recent evidence furthermore suggests that the assessment of hemodynamic forces indicates impairment of LV systolic ejection force in HFpEF which is associated with CVH. Whether or not this will provide an accurate clinical parameter of systolic impairment in HFpEF beyond volumetric or strain-based indices of systolic function will yet have to be investigated.^72^

Nevertheless, as previously shown, exercise stress is of great importance for the diagnosis of HFpEF. Novel real time CMR techniques allow the acquisition of individual heart beats with high resolution in real time without the need to average cardiac function over several heart beats. The proposed techniques facilitate a quantitative assessment of diastolic and systolic myocardial function using physiological exercise CMR.^73^ Recently, the HFpEF-Stress trial could show that exercise CMR is capable of a highly accurate identification of HFpEF after comparing it to the invasive reference standard of RHC.^74^ In particular left atrial long axis shortening (LA-LAS) during exercise stress was proven to be a strong and independent predictor of HFpEF.^74^ This offers a non-invasive alternative to stress-echocardiography, as CMR is less sensitive to patients’ constitution.

#### Calculation of arterial stiffness

Since arterial stiffness as a decrement of vessel compliance was observed to be strongly associated with echocardiographic parameters predicting diastolic dysfunction^75^ it is receiving rising interest. Diastolic dysfunction and increased arterial stiffness share most risk factors like chronic inflammation, aHT and diabetes.^76^ Additionally, the ventricular–vascular interaction of vascular stiffness with increased pulse pressure and LV afterload may accelerate the progression of HFpEF.^77^^,^^78^ This is supported by the correlation of invasively measured arterial stiffness with abnormally steep increases of PCWP during exercise.^79^ Interestingly, this effect was stronger in women who are known to have a higher prevalence of HFpEF.^1^ However, the actual contribution of arterial stiffness to the development of HFpEF remains unclear.^79^

**Fig. 5:**
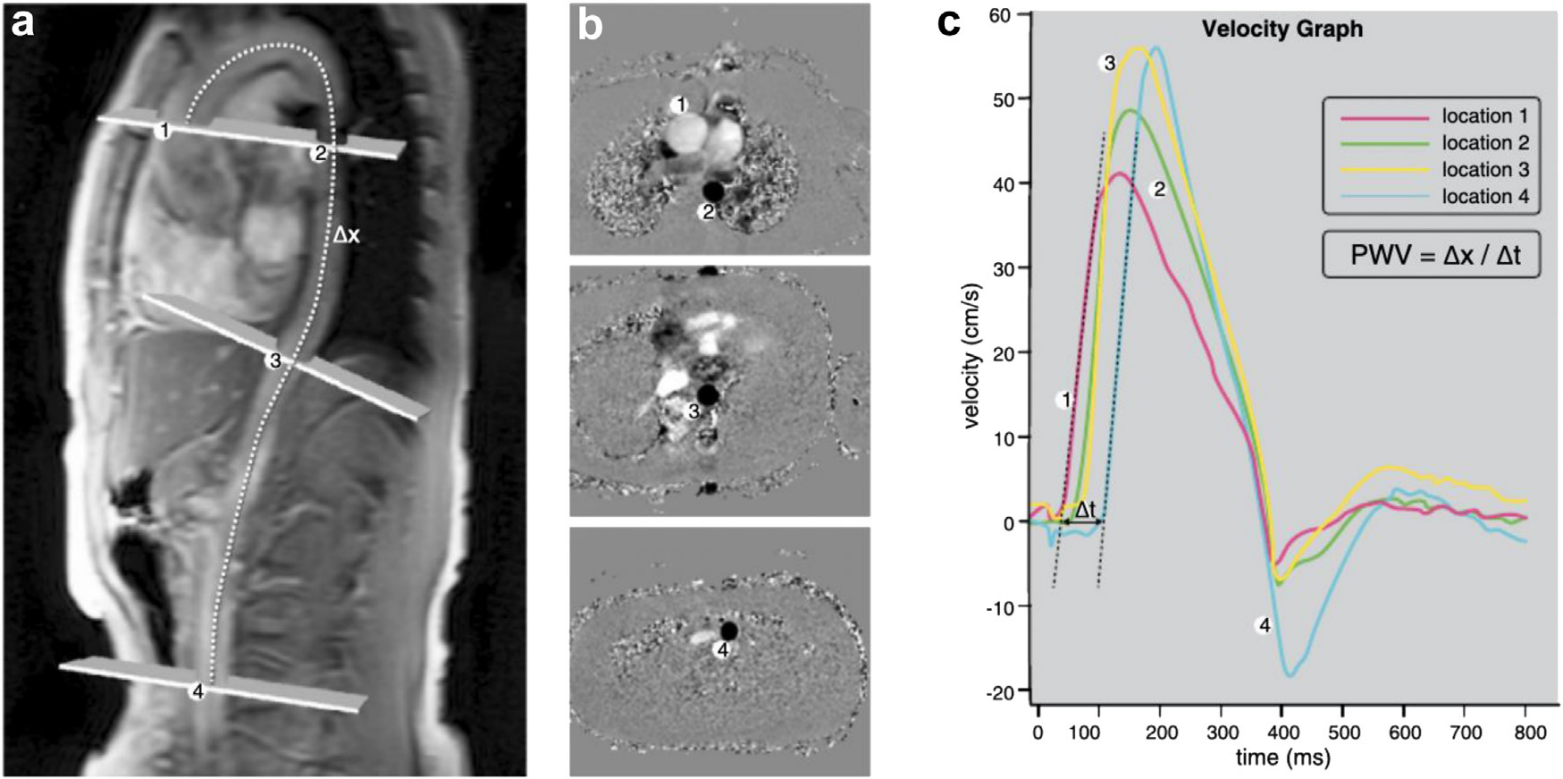
Calculation of aortic pulse-wave-velocity (PWV) with CMR. (a) Slice positioning alongside the aortic arch with the dotted line highlighting the centerline across the aorta for later calculation of the PWV. (b) Inplane views of the corresponding phase-contrast velocity images for blood flow calculations. (c) PWV is calculated with the distance (Δx) measured along the center line and the transit-time (Δt) of the velocity wave forms down the aortic arch (van Hout et al. 2021,^81^ © CC-BY 4.0 https://creativecommons.org/licenses/by/4.0/).

As CMR phase-contrast flow-measurements can be used for a comprehensive calculation of pulse-wave-velocity (see Fig. 5) over the aorta as a marker of arterial stiffness,^80^ its quantification seems promising for further investigation of a potentially causative factor to HFpEF.

#### 4D-flow imaging

**Fig. 6:**
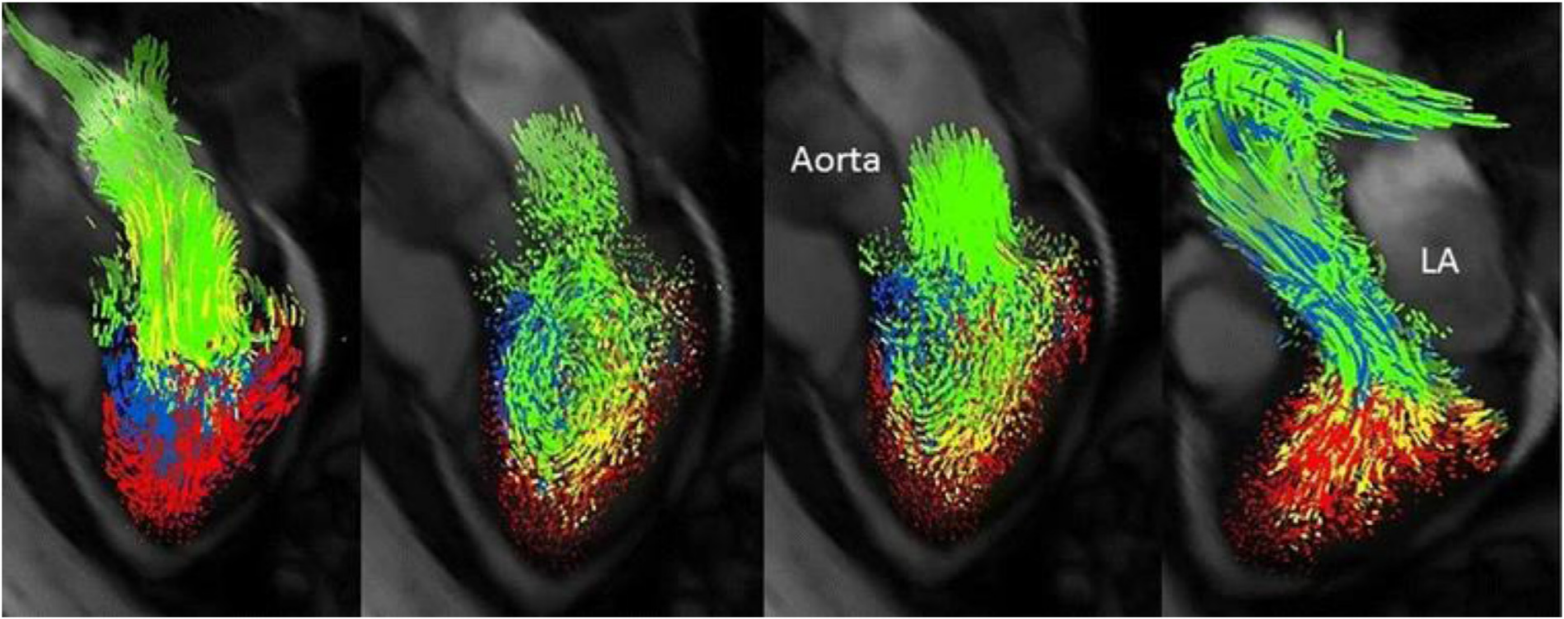
Example for visualization of left ventricular blood flow components. Displayed are the four phases of the cardiac cycle from left to right: early diastole, diastasis, atrial contraction, systole. Green marks the direct flow, yellow is the retained inflow, blue highlights the delayed injection flow and red the residual volume. LA = Left atrium. Modified after Stoll et al. 2018,^90^ © CC-BY 4.0 https://creativecommons.org/licenses/by/4.0/.

By the quantification of phase shifts in a specific phase-encoding direction, CMR measures blood flow velocity and absolute volumes (Fig. 6).^82^ By augmenting the phase encoding into three directions of space over time, 4D-flow measurements and visualizations of kinetic energies can be derived.^83^^,^^84^ While 4D-flow measurements involved time-consuming post-processing in the past, recent advances using accelerated imaging techniques such as compressed sensing, showed promising results for its future usability.^85^ First implementations for 4D-flow quantification in cardiac CT have been made as well but require further technical development and validation.^86^

Intracardiac 4D-flow measurements were able to deliver deeper insights to patients with conventional heart failure and the prediction of their exercise capacity. In particular, in patients with dilated or ischemic cardiomyopathy, the direct kinetic energy was shown to be an independent predictor of the functional capacity and could be used for therapy monitoring and prognostication in the future.^87^

In patients with atrial fibrillation despite of similar LV mass, volume and EF; 4D-flow measurements were able to indicate that patients suffering from AF had reduced direct flow and a delayed ejection compared to controls.^88^ As AF is strongly associated with HFpEF this might offer a chance to understand the kinetics of the failing ventricle and patterns of remodeling with changes in contraction and relaxation of the ventricle.

Albeit this method needs further validation, its calculation can be performed semi-automatically with low inter- and intraobserver variability and independently from potential confounders like the angulation in TTE.^89^

### Cardiac perfusion, metabolism, and innervation

#### Microvascular disease and coronary venous congestion

While the role of the great arteries in HFpEF has been described above, the importance of coronary vessels should be equally emphasized. Coronary artery flow is known to mainly occur during diastole of the cardiac cycle. The impaired lusitropy in HFpEF may reduce coronary artery flow and subsequently myocardial perfusion, which in turn is highly likely to accelerate myocardial remodeling followed by diastolic dysfuntion.^91^ Studies have already shown that the myocardial perfusion reserve in patients with HFpEF is significantly lower compared to controls^92^ and that microvascular disease in patients with a high burden of risk factors and precursors to HFpEF is independently associated with the development of HFpEF.^93^

**Fig. 7:**
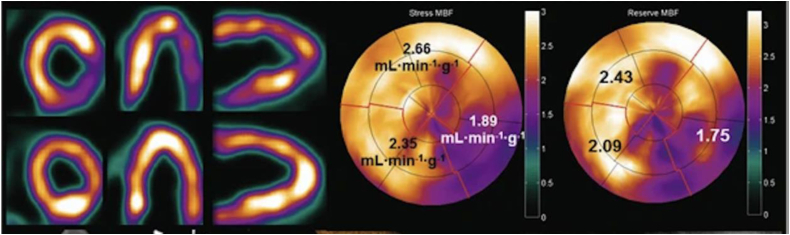
PET-derived perfusion imaging. This example displays an inferolateral perfusion defect with corresponding bulls-eye maps of myocardial perfusion under stress and the calculation of the reduced myocardial flow reserve. While this technique is mostly used for coronary artery disease, its capabilities can be transferred to image microvascular disease in HFpEF as well. MBF = Myocardial blood flow. Modified after Driessen et al. 2017,^94^ © CC-BY 4.0 https://creativecommons.org/licenses/by/4.0/.

Microvascular disease can be diagnosed by a reduced coronary flow reserve with PET-CT being a well-established non-invasive test for this diagnosis. Using PET and distinct tracers, it is possible to accurately quantify myocardial blood flow and the myocardial perfusion reserve (Fig. 7).^94^ As an alternative, CMR with contrast-enhanced stress perfusion imaging also yields the capabilities for an accurate identification and quantification of myocardial perfusion with even higher resolution compared to PET-CT.^95^, ^96^, ^97^ Direct head to head comparisons revealed good correlation and a high accuracy of both imaging modalities when diagnosing coronary artery disease. However, as absolute perfusion values revealed discrepancies, further validation is required.^98^

On the other end of coronary flow, coronary venous congestion was found to be another important player in HFpEF.

Pulmonary arterial hypertension or pulmonary oedema with subsequently increased RA filling pressure can result in coronary venous congestion, which is known to cause myocardial edema.^99^^,^^100^ As the edema compromises myocardial function and induces adverse remodeling^101^ this could play an important role in the development and progression of diastolic dysfunction.

In clinical imaging, CMR tissue characterization with T2-based imaging and mapping of myocardial edema was strongly associated with hypertrophy and diastolic dysfunction.^102^ As this parameter is often derived during routine CMR it should receive rising attention and might help to identify patients at risk for HFpEF before remodeling has begun.

#### Cardiac metabolism and innervation

Cardiac edema is a marker for inflammation and can be assessed using T2-weighted imaging in CMR as explained previously. While edema is just an indirect measure of inflammation, FDG-PET can differentiate small changes in myocardial metabolism. While the atria cannot be sufficiently assessed by CMR T2 mapping, PET is able to identify focal regions in the atria with increased metabolism as a sign of inflammation.^103^^,^^104^ The atrial metabolism can be considered to be from special interest as the atria directly contribute to diastolic dysfunction and their remodeling due to the pro-inflammatory state in HFpEF is rather unclear.

More experimental techniques enable direct imaging of the inflammation in the heart by additional use of hyperpolarized contrast agency in CMR^105^ or ^64^Cu-macrin in PET^106^ to visualize macrophages. However, those techniques require further validation before they can be applied in a clinical environment.

Besides cardiac metabolism, increased cardiac innervation by the sympathetic nerve system with an increased adrenergic drive was found to accelerate cardiac remodeling.^107^^,^^108^ This was not only an important mechanism of the progression of heart failure and outcome prediction in patients with HFrEF^109^ but also contributes to the development of diastolic dysfunction in patients with arterial hypertension.^110^ Using Cardiac ^123^I-MIBG SPECT to determine sympathetic presynaptic nerve function, a correlation between the severity of diastolic dysfunction and remodeling was found.^111^ Interestingly, cardiac sympathetic nerve dysfunction is even able to predict future cardiac events.^112^ While this pathophysiological mechanism is hard to study with other non-invasive imaging techniques it might be underrecognized currently but could be of great interest in the future.

### Extracardiac structures

#### Adipose tissue quantification

**Fig. 8:**
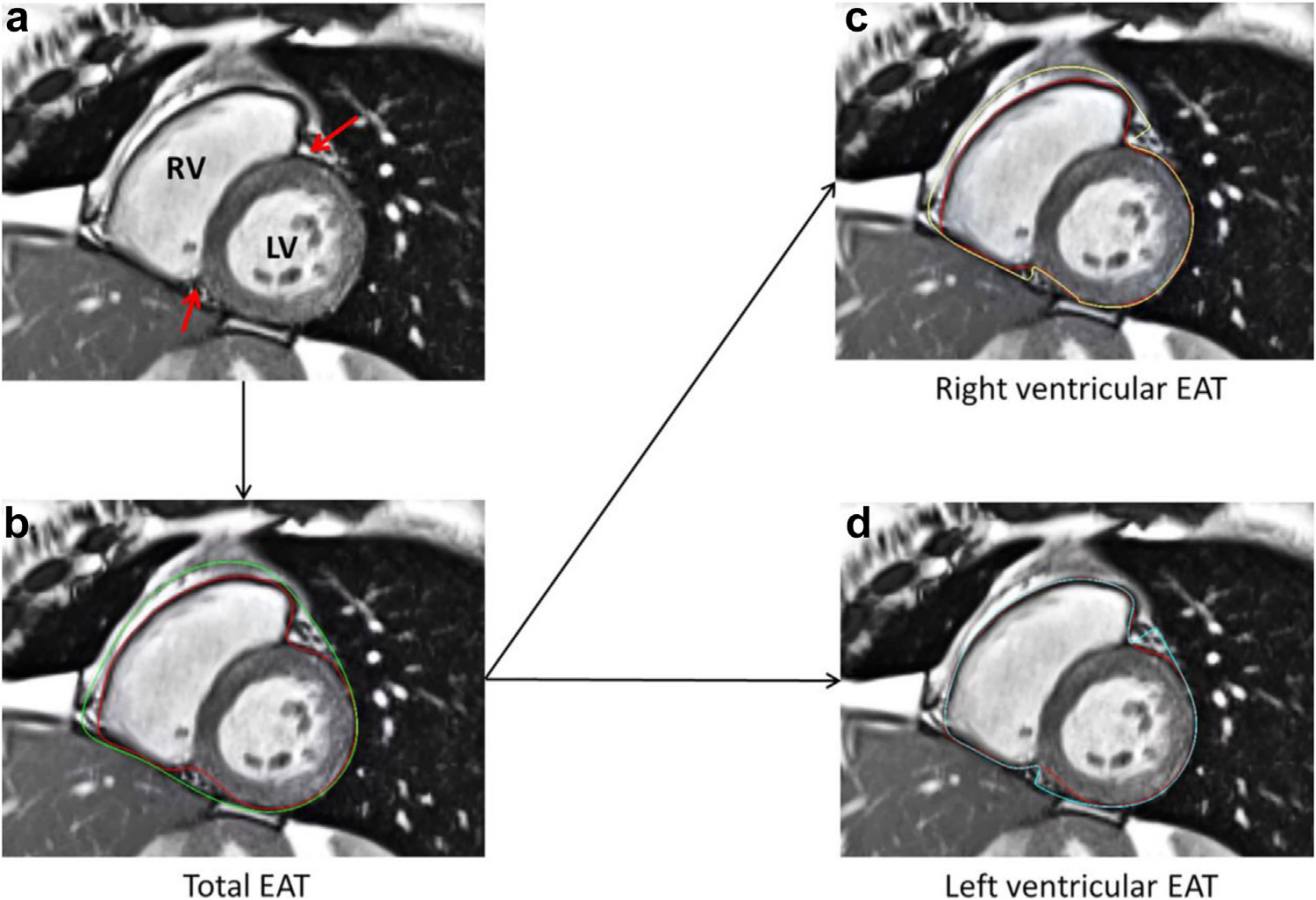
Quantification of regional epicardial adipose tissue (EAT) in CMR. (a) A midventricular short-axis view of the left (LV) and right ventricle (RV) is shown. Red arrows mark the anterior and posterior insertion points. (b) Calculation of the total EAT between the epicardium (red line) and pericardium (green line). (c) Calculation of the RV-EAT. (d) Calculation of the LV-EAT (van Woerden et al. 2021,^114^ © CC-BY 4.0 https://creativecommons.org/licenses/by/4.0/).

As mentioned, adipose tissue has an important role as a substrate and risk factor for diastolic dysfunction and HFpEF.^16^ While the systemic pathway comprising an inflammatory process has been described, the local impact of adipose tissue next to the heart i.e. epicardial fat, is not completely understood. Initial studies found an association between obesity with increased right ventricular epicardial adipose tissue (EAT) and higher right-sided filling pressure as well as lower exercise capacities in patients suffering from HFpEF.^113^ This observation led to more detailed investigations of the EAT and its distribution around the heart in affected patients (see Fig. 8). In particular, regional EAT was observed to impact local cardiac structure^114^ which strengthens the hypothesis of its direct involvement in the pathophysiology. Recent studies observed adverse outcomes in patients with HFpEF and increased EAT.^115^ Those findings are supporting the important role of EAT in HFpEF and suggesting that a detailed assessment may facilitate future targeted therapies.

#### Lung water density

Another important adjunct is the visualization of the pulmonary congestion causing exertional dyspnea as one of the first symptoms in patients with HFpEF.^116^

Transit pulmonary congestion could be observed during submaximal exercise stress already, and was linked to impaired exercise hemodynamics in patients with HFpEF.^117^^,^^118^ It is proposed that an energy deficit during exercise might be a key factor to the pulmonary congestion,^119^ while the increased lung water is a consequence to increased cardiac filling pressure in HFpEF.^120^

**Fig. 9:**
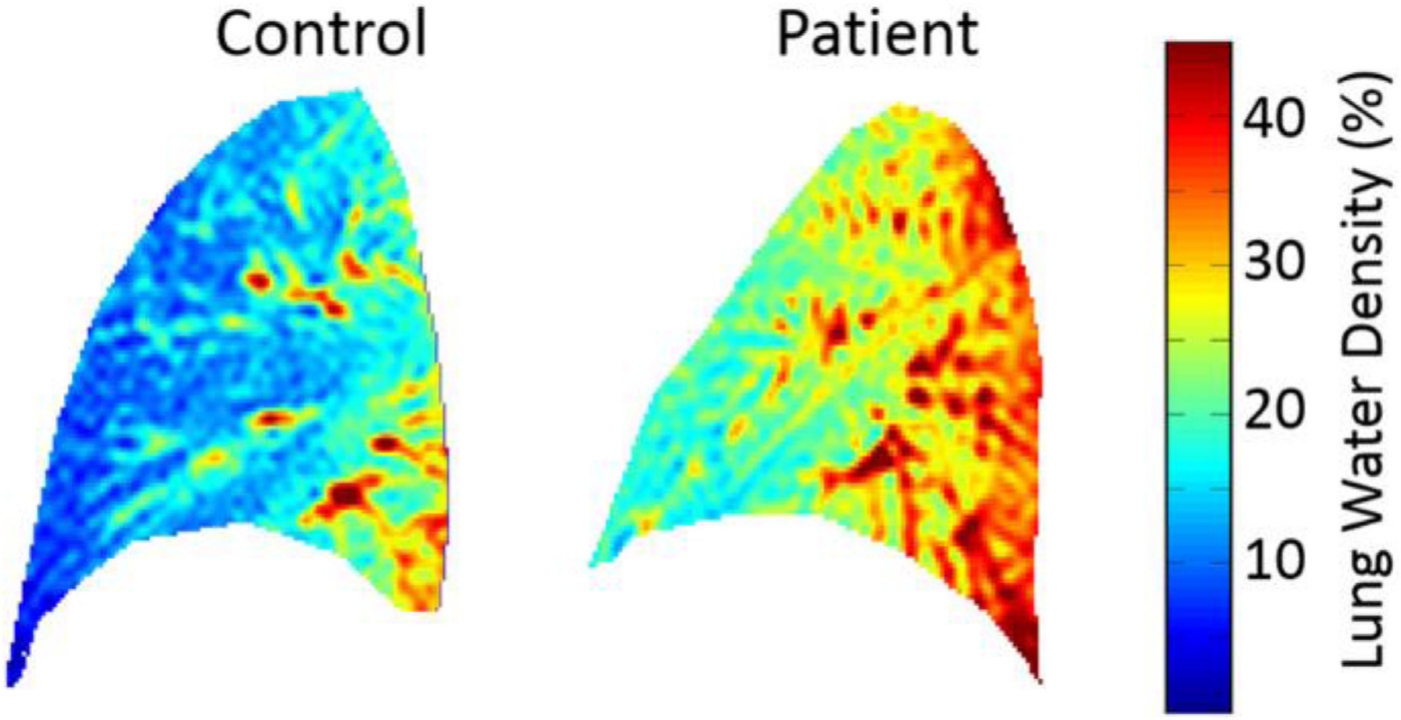
Visualization of lung water density with CMR. Displayed is the lung water density of a healthy control vs. a patient with heart failure. The lung water density was 16.5% in the control and 27.1% in the patient respectively. The patient was observed to have an increased left ventricular end diastolic pressure and brain natriuretic peptide at the same time.^121^ (Thompson et al. 2019,^121^ CC-BY 4.0 https://creativecommons.org/licenses/by/4.0/).

Using proton density maps, CMR allows for a straightforward quantification of lung water density (see Fig. 9) and qualifies for risk stratification as increased lung water density emerged as an independent predictor for death, hospitalization or emergency department visit within one year in HFpEF.^121^ Combining this technique with CMR-compatible exercise stress testing it could shed light on the differing extend of dyspnea in patients with HFpEF and lead to individualized therapy approaches.

## Prognostic implications

HFpEF and HFrEF were observed to have similar outcomes regarding hospitalization, symptomatic burden and quality of life.^19^^,^^122^ However, the prognosis in patients with HFpEF is barely described. While hospitalized patients have been shown to have equivalent mortality rates^19^ a large community-based study has found a lower mortality in patients suffering from HFpEF compared to HFrEF in general.^123^ It is speculated that HFpEF is over-diagnosed in the group at risk. In those patients, diastolic dysfunction is common, and associated echocardiographic abnormalities in combination with non-specific clinical symptoms or deconditioning may lead to diagnostic errors.^124^ While independent predictors of mortality have already been defined including patient characteristics, laboratory markers and echocardiographic parameters,^9^ the latter suspicion strengthens the need for a more detailed non-invasive characterization in patients at risk. CMR has been shown to have the capabilities for prognostication in patients using distinct imaging parameters^125^^,^^126^ and various other sophisticated multiparametric imaging techniques and markers are emerging for an enhanced risk stratification and outcome prediction.

### Outstanding questions

Innovative techniques may pave the way for an enhanced characterization of a complex pathophysiology; however, they will have to undergo further validation and the clinical benefit has yet to be proven. Some of the techniques are very complex in their everyday application and require highly specialized centers to use and interpret them correctly. The simplification and standardization is an important area of ongoing research.

In clinical routine, diagnostic markers are indispensable for the correct description of a patients’ medical condition. Further studies will need to focus on various aspects while using innovative imaging markers. This includes reproducibility, cost efficiency and efficiency of use, while they must be closely related to the pathophysiology and ease diagnostic decision making to identify patients at risk and improve their outcomes.

## Conclusion

The non-invasive diagnosis of HFpEF is challenged by the complexity of the disease in clinical practice. While modern techniques like speckle-tracking for echocardiography enhance the monitoring of the LV contraction and relaxation, other imaging modalities like CMR, CT or PET-CT are quickly evolving. By using the combined capabilities of myocardial tissue characterization and functional quantification at rest and during exercise, CMR yields high diagnostic value for the diagnosis of HFpEF. Recent developments promise more insights into the complex pathophysiology of this condition, a better risk stratification, and better therapeutic guidance.

## Search strategy

PubMed and relevant articles from 1990 to 2022 were screened for references to this article. We included the search terms: “HFpEF”, “HFpEF diagnosis”, “HFpEF imaging”, “Diastolic dysfunction”, “Heart failure with preserved ejection fraction”, “HFpEF non-invasive diagnosis” and “HFpEF pathophysiology”. The final references were chosen based on their relevance to the main topic of the article and their importance to current diagnostic guidelines and innovations. All references were discussed by the authors before their final use.

## Contributors

Both authors wrote, reviewed, and edited this manuscript. Both authors read and approved the final version of the manuscript.

## Declaration of interests

None.
